# The relationship between birch pollen, air pollution and weather types and their effect on antihistamine purchase in two Swedish cities

**DOI:** 10.1007/s10453-017-9478-2

**Published:** 2017-04-06

**Authors:** Maria Grundström, Åslög Dahl, Tinghai Ou, Deliang Chen, Håkan Pleijel

**Affiliations:** 1 Department of Biological and Environmental Sciences, University of Gothenburg, P.O. Box 461, 405 30 Gothenburg, Sweden; 2 National Pollen and Aerobiological Research Unit, Institute of Science and the Environment, University of Worcester, Henwick Grove, Worcester, WR2 6AJ UK; 3 Department of Earth Science, University of Gothenburg, P.O. Box 460 405 30, Gothenburg, Sweden

**Keywords:** Pollen, *Betula*, Birch, Air pollution, Weather type, Antihistamine

## Abstract

Exposure to elevated air pollution levels can aggravate pollen allergy symptoms. The aim of this study was to investigate the relationships between airborne birch (*Betula*) pollen, urban air pollutants NO_2_, O_3_ and PM_10_ and their effects on antihistamine demand in Gothenburg and Malmö, Sweden, 2006–2012. Further, the influence of large-scale weather pattern on pollen-/pollution-related risk, using Lamb weather types (LWTs), was analysed. Daily LWTs were obtained by comparing the atmospheric pressure over a 16-point grid system over southern Sweden (scale ~3000 km). They include two non-directional types, cyclonic (C) and anticyclonic (A) and eight directional types depending on the wind direction (N, NE, E…). Birch pollen levels were exceptionally high under LWTs E and SE in both cities. Furthermore, LWTs with dry and moderately calm meteorological character (A, NE, E, SE) were associated with strongly elevated air pollution (NO_2_ and PM_10_) in Gothenburg. For most weather situations in both cities, simultaneously high birch pollen together with high air pollution had larger over-the-counter (OTC) sales of antihistamines than situations with high birch pollen alone. LWTs NE, E, SE and S had the highest OTC sales in both cities. In Gothenburg, the city with a higher load of both birch pollen and air pollution, the higher OTC sales were especially obvious and indicate an increased effect on allergic symptoms from air pollution. Furthermore, Gothenburg LWTs A, NE, E and SE were associated with high pollen and air pollution levels and thus classified as high-risk weather types. In Malmö, corresponding high-risk LWTs were NE, E, SE and S. Furthermore, occurrence of high pollen and air pollutants as well as OTC sales correlated strongly with vapour pressure deficit and temperature in Gothenburg (much less so in Malmö). This provides evidence that the combination of meteorological properties associated with LWTs can explain high levels of birch pollen and air pollution. Our study shows that LWTs represent a useful tool for integrated daily air quality forecasting/warning.

## Introduction

Pollen allergy affects up to 40% of the population in Northern Europe (d’Amato et al. 2007). In South Sweden, birch (*Betula*) pollen, along with grass (Poaceae) pollen, is the main cause. In a study, assessing the situation in west Sweden from 2012, 29% of the population aged 20–46 was sensitized to grass and 24% to birch allergens (Bjerg et al. 2016). Allergy sufferers are not only influenced by allergens. Epidemiologic and experimental studies show that health effects arise from simultaneous exposure to pollen and air pollution (Ghosh et al. 2010; Peden and Reed 2010). Nitrogen dioxide (NO_2_) and particulate matter (PM) are linked to effects on the respiratory and cardiovascular systems (Samoli et al. 2006; Chiusolo et al. 2011). Air pollutants act as irritants and can induce airway inflammation and disruption of epithelial barrier homeostasis, which facilitates the access of allergens to effector cells of the allergic immune response (Traidl-Hoffman et al. 2009). Ozone, NO_2_ and PM_10_ induce the production of reactive oxygen species, which aggravate allergic inflammation (Klein et al. 2012). Hence, it is important to understand conditions for coincident high exposure to pollen and air pollution, and to what degree pollutants contribute to airway symptoms. Such information is useful in forecasting and early warning systems integrating air pollution and pollen.

The readiness to flower in birch is governed by temperature over several months (Dahl et al. 2013). When the critical temperature sum is achieved, short-term meteorological conditions determine pollen release, atmospheric concentration and transport (Faegri and Iversen 1989; Dahl et al. 2013; Sofiev et al. 2013). Pollen release is due to dehydration of the anther wall (Pacini and Hesse 2004), which depends on the drying power of the air, reflected by the vapour pressure deficit, VPD. Often, relative humidity, RH, is used as an indicator of air dryness. However, the drying power at a certain RH is strongly dependent on temperature, which may lead to misleading conclusions when comparing situations differing in temperature. VPD on the other hand is a direct measure of the drying power of the air, independent of temperature (Campbell and Norman 1998). One could therefore expect processes depending on the drying power of the air to be more strongly linked to VPD than to RH. Furthermore, solar radiation and low to moderate wind speeds favour dehydration of the anther wall and pollen release also depends on a temperature-dependent maturation process, causing active retraction of water (Dahl et al. 2013). Wind will promote pollen release from the boundary layer of the tree and assist transport.

Pollen dispersion ranges from microscale to the continental scale. Most pollen is deposited near the source, but a small fraction travels very far (Faegri and Iversen 1989; Campbell et al. 1999; Sofiev et al. 2013; Šikoparija et al. 2013). At a local scale, low to moderate wind speeds (2–4 m s^−1^) are optimal to create high pollen concentrations at ground level (Sofiev et al. 2013). Stronger winds will reduce pollen concentrations due to dilution (Makra et al. 2006; Khwarahm et al. 2014). Dry deposition by impaction or sedimentation removes pollen from the atmosphere. Wet deposition during precipitation strongly reduces pollen concentrations (Sofiev et al. 2013).

Air pollutant levels are also strongly related to meteorology. Low wind speed and stable atmospheric conditions are important for high levels of NO_2_ (Grundström et al. 2015a), while for PM both high and low wind speed can be associated with high levels (Jones et al. 2010; Grundström et al. 2015b). Ground-level ozone is a secondary pollutant. Its formation depends on NO_2_, volatile organic compounds and solar radiation. Hence, high levels of ozone are often associated with calm anticyclonic conditions (Tang et al. 2009). Thus, it can be assumed that certain sets of meteorological conditions (calm, dry, warm) simultaneously promote high levels of both pollen and air pollutants, which may lead to an aggravation of respiratory symptoms.

Relationships between a single and a small suite of meteorological variables, e.g., temperature, relative humidity, sunshine hours, rainfall and wind speed/direction and atmospheric pollen concentrations, are well established (Schäppi et al. 1997; Khwarahm et al. 2014). However, plants respond to the combination of factors prevailing in a certain weather situation, not to factors one-by-one. A partitioning of weather conditions into weather types based on atmospheric pressure is a practical way to aggregate the complexities of weather patterns into relevant categories (Dixon et al. 2016). Such a classification provides a framework in which the link between meteorological conditions with pollen and/or air pollution can be effectively investigated. So far, few studies along this line have been reported (Laiidi 2001; Makra et al. 2006; Makra et al. 2015; Hebbern and Cakmak 2015). The Lamb weather type (LWT) system is a weather classification scheme which describe the synoptic atmospheric circulation exhibiting either a directional or circulatory (vorticity) air movement. By identifying the air mass flow over a given region and temporal scale, LWTs provide a means of summarizing meteorological conditions, which can be very specific for each LWT. The LWT system has been successfully used in Europe to study impacts of weather on a number of phenomena (Chen 2000; Tang et al. 2009; Demuzere et al. 2009; Grundström et al. 2015a).

Over the last few years, the relationship between health parameters and short-term effects of airborne pollen concentration has been in focus for epidemiological studies (Lierl and Hornung 2003; Dales et al. 2004; Feo Brito et al. 2007; Ghosh et al. 2010). They have been dominated by studies of the most severe reactions, i.e., the number of asthma exacerbations resulting in visits to emergency rooms and hospital admissions. Less dramatic symptoms attracted less attention, although they have a considerable effect on quality of life, production loss and societal costs. They can be measured using proxies such as over-the-counter (OTC) sales of antihistamines (e.g., Fuhrman et al. 2007; Motreff et al. 2014; Caillaud et al. 2015; Sheffield et al. 2011). These drugs are commonly used for a range of allergic manifestations. The OTC data provide information on the entire population to a low cost, albeit this information is an indirect measure of symptom severity, which is affected by the behaviour of the allergy sufferers.

The aim of our study was to investigate the influence of synoptic weather, represented by LWTs, on atmospheric concentrations of birch pollen and air pollution ([NO_2_], [PM_10_], [O_3_]) in two Swedish cities, Gothenburg and Malmö. We attempted to identify weather types linked to a risk of high birch pollen and air pollution levels individually and simultaneously and to determine whether these situations were linked to enhanced sales of over-the-counter (OTC) antihistamines.

Two hypotheses were tested:

1. High exposure situations of birch pollen and air pollutants occur during weather types associated with warm, dry and moderately calm air masses.
2. Simultaneous high exposure to birch pollen and air pollutants generate an increase in the number of OTC antihistamine doses sold.

## Materials and methods

### Data and measurement sites

#### Pollen data

Pollen data from the years 2006–2012 were obtained from the Pollen Laboratory, University of Gothenburg for both Gothenburg and Malmö, using Burkard 7-day recording volumetric spore traps of the Hirst design (Hirst 1952). In Gothenburg, pollen is monitored on a rooftop, 40 m above ground level, at Sahlgrenska University Hospital “Östra” in the eastern part of Gothenburg city (57°43.34′N, 12°3.12′E). The area is surrounded by residential areas, woodlands in the east and south, and urban ground to the west. In Malmö, pollen is monitored on a rooftop 30 m above ground at Skåne University Hospital, in the southern part of the city centre (55°35.38′N, 13°0.11′E). The area is mainly surrounded by urban ground with a large park to the west. The birch pollen season was considered to start when atmospheric birch pollen was recorded for more than three consecutive days, and it was considered to have ended when zero birch pollen was recorded for more than three consecutive days. On average, the birch pollen season lasted for approximately two months starting in early April and ending in early June.

Birch pollen is the dominant aeroallergen in South Sweden during the second half of April and the beginning of May. There may be some noise in the data from the contributions of a few other allergenic pollen types, viz. from beech, oak and grass pollen. Beech, which is on the northern border of its distribution in South Sweden, does not flower every year and peaks with high values that occur only occasionally. The start of grass and oak anthesis overlaps with the end of birch flowering and can be responsible for some of the antihistamine demand, however, only during a minor part of the birch pollen season, and not every year. Other allergenic pollen types of significance occur earlier or later than the birch pollen season. Airborne fungal spores may have an influence; however, less than 2% of the population are sensitized to fungi (Bjerg et al. 2016).

#### Air pollution and meteorological data

In Gothenburg, monitoring of air quality and meteorological data was performed on a rooftop 30 m above ground level in the commercial district of the city (“Femman”; 57°42.52′N, 11°58.23′E). The site is located adjacent to the central terminal for busses and trains and approximately 300 m away from a busy traffic route (highway E45). Hourly measurements of NO_2_ (Tecan CLD 700 AL chemiluminescence instrument), PM_10_ (Tapered Element Oscillating Microbalance, Series 1400b), atmospheric pressure (Vaisala PA11A), air temperature and relative humidity (Campbell Rotronic MP101 thermometer/hygrometer), wind direction and wind speed (Gill ultrasonic anemometer) were taken.

In Malmö, air quality was measured on a rooftop 20 m above ground (“Rådhuset”; 55°36.38′N, 13°0.11′E) and meteorological variables were measured just a few blocks away to the east of the Skåne University Hospital (“Heleneholm”; 55°34.28′N, 13°4.41′E). For Malmö, atmospheric pressure and radiation data were missing.

For the analysis, hourly time resolution data were converted into daily time resolution (further details in Sect. 2.3).

#### Allergy medication data

In Sweden, all dispensed pharmaceuticals, either prescribed or purchased over-the-counter (OTC), have to be reported electronically to national registers, and data are available for research to a small administrative cost. When the Swedish pharmacy monopoly was first abolished in 2009, OTC sales were registered by “Apotekens Service AB”, a company co-owned by all pharmacies. All daily purchased antihistamines (measured as “defined daily dose”) to men and women within the age span 10–64 years, attributed to the ATC-codes R01AC01, R01AC02, R01AD01, R01AD02, R01AD05 and R01AD09, were included, in the cities Malmö and Gothenburg, during the birch pollen season for 2006–2012. All data are anonymous in terms of personal data.

### LWTs

Daily mean sea level pressure (MSLP) for 16 grid points centred over the Gothenburg city centre (57°7′N, 11°97′E) and Malmö city centre (55°59′N, 13°01′E) was obtained from the NCEP/NCAR Reanalysis database 2.5 × 2.5 degree pressure fields (Kalnay et al. 1996). Circulation indices, *u* (westerly or zonal wind), *v* (southerly or meridional wind), *V* (combined wind speed), *ξ*_u_ (meridional gradient of *u*), *ξ*_v_ (zonal gradient of *v*) and *ξ* (total shear vorticity), describing the geostrophic winds and LWTs (Jenkinson and Collison 1977) were calculated following Chen (2000). The classification scheme has 26 weather types: anticyclone (A), cyclone (C), 8 directional types (NE, E, SE, …) and 16 hybrid types (ANE, AE, ASE, CNE, CE, CSE, …). In this study, the 26 weather types were consolidated into 10 LWTs according to the directions of the geostrophic wind, eight directional: NE, E, SE, S, SW, W, NW, N, and two rotational: A and C. LWTs were analysed for the birch pollen seasons of 2006–2012.

### Calculations

For each LWT, averages for the concentrations of birch pollen, air pollutants and meteorological variables, were calculated. VPD was calculated following Teten’s formula (Campbell and Norman 1998);

$${\text{VPD}} = e_{\text{s}} \left( {T_{\text{a}} } \right) - e_{\text{a}} = e_{\text{s}} \left( {T_{\text{a}} } \right)\left( {1 - h_{\text{r}} } \right)$$

where *e*_s_ is the saturation vapour pressure at a given temperature, *T*_a_, is the ambient air temperature, *e*_a_ is the vapour pressure at a given temperature, and *h*_r_ is the relative humidity. Birch pollen levels are represented by daily sums. When this sum exceeds 100 grains m^−3^, the level is defined as high, as is the practice in Sweden. This threshold was introduced to the national pollen warning system in the 1970s and is supposed to reflect the severity of symptoms for the average allergy sufferer, with reference to “clinical experience”. Time fractions of these threshold level exceedances were then calculated for each LWT. Time fractions express the fraction of total number of days in which a certain criterion was fulfilled with unit  %. For air pollutants, a daily maximum was calculated from each 24 h daily sets. A threshold exceedance was determined valid if the daily maximum concentration exceeded the threshold values ([NO_2_]_max_ > 60 µg m^−3^, [PM_10_]_max_ > 50 µg m^−3^ and [O_3_]_8h max_ > 80 µg m^−3^). Then, the time fractions of these exceedances within the respective LWTs were calculated for each pollutant. The threshold values for PM_10_ and NO_2_ are motivated by current Swedish air quality legislation and are based on the existing daily threshold values. The threshold for ozone is a Swedish environmental quality objective for clean air. This way of calculating does not reflect an actual exceedance of the specific air quality standard or environmental objective, but in this study the threshold exceedances are used as an estimate/definition of days reaching high levels of air pollutants.

Correlation analysis was conducted to understand whether situations with simultaneous exposure to high levels of birch pollen and to pollutants (NO_2 max_, PM_10 max_ or O_3 8h max_) were more common in some LWTs than in others. Furthermore, analysis of the correlation between frequencies of high exposure and meteorological variables among LWTs was carried out. We also studied the effect of concurrent high birch pollen and high air pollution levels on OTC sales of antihistamines, as compared to the effect of high birch pollen alone. The association between the variables in all relationships was analysed using the Pearson product moment correlation coefficient (*r*), and all variables were tested for normality using the Shapiro–Wilk test. Statistical significance (*p* value < 0.05) was determined using the *F*-test with the null hypothesis of a none-existent relationship (slope = 0). All statistics were carried out using relevant functions included in the *R* Stats Package.

## Results and discussion

### Meteorological characteristics under LWTs during the birch pollen season

**Fig. 1:**
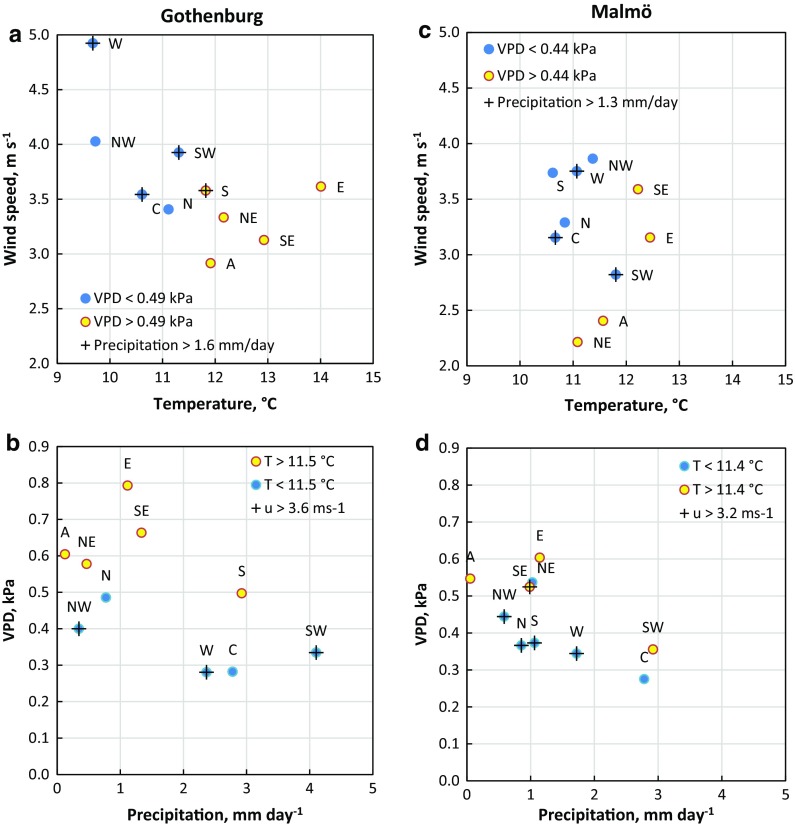
Scatter plots showing the average temperature, wind speed, precipitation and VPD for LWTs during the pollen season in Gothenburg (**a**, **b**) and Malmö (**c**, **d**). In **a** and **c**, *blue circles* signify VPD below the average, *yellow circles* signify VPD above the average and *plus symbols* signify precipitation above the average. In **b** and **d**, *blue circles* signify temperature (T) below the average, *yellow circles* above the average and *plus symbols* signify above average wind speeds. Averages of meteorological variables are specific for each city and shown in Table 1

In Gothenburg, the LWTs were distinct and differed quite clearly from one another with regard to meteorological characteristics (Table 1 and Fig. 1a, b). Two semi-coherent LWT groups were distinguishable for Gothenburg based on VPD and precipitation (Fig. 1b). Six of the ten LWTs stood out, where the average situation was dry (high VPD), warm (apart from N and NW) and with low precipitation, namely LWTs A, NE, E, SE, N and NW (Table 1). They were all associated with high atmospheric pressure (>1010.8 hPa, Table 1) apart from NW. The remaining LWTs were associated with low atmospheric pressure and were generally cooler and windier (especially LWTs W, NW and SW). High precipitation was associated with LWTs S, SW, W and C.

**Table 1:** Number of days (*n* days) and averages of meteorological variables for LWTs in Gothenburg (G) and Malmö (M) during the birch pollen season

LWT	*n* days LWT		*P* (hPa)		RH (%)		*T* (°C)		*u* (m s^−1^)		*R* (W m^−2^)		Precip. (mm day^−1^)		VPD (kPa)	
	G	M	G	M	G	M	G	M	G	M	G	M	G	M	G	M
A	107	94	1019.7	–	63.5	66.9	11.9	11.6	2.9	2.4	237.3	–	0.1	0.1	0.60	0.55
NE	22	21	1014.5	–	63.0	69.9	12.2	11.1	3.3	2.2	216.3	–	0.5	1.0	0.58	0.54
E	41	41	1016.1	–	53.6	65.9	14.0	12.5	3.6	3.2	212.5	–	1.1	1.1	0.79	0.60
SE	25	29	1011.9	–	62.5	70.9	12.9	12.2	3.1	3.6	180.2	–	1.3	1.0	0.66	0.52
S	30	27	1007.9	–	70.3	75.3	11.8	10.6	3.6	3.7	152.5	–	2.9	1.1	0.50	0.37
SW	33	32	1006.8	–	77.7	78.0	11.3	11.8	3.9	2.8	144.5	–	4.1	2.9	0.33	0.36
W	51	37	1008.3	–	78.3	76.3	9.7	11.1	4.9	3.8	157.6	–	2.4	1.7	0.28	0.34
NW	65	40	1009.6	–	68.8	71.3	9.7	11.4	4.0	3.9	217.2	–	0.3	0.6	0.40	0.44
N	37	25	1011.9	–	67.0	74.6	11.1	10.8	3.4	3.3	226.0	–	0.8	0.9	0.49	0.37
C	47	38	1001.6	–	79.7	81.3	10.6	10.7	3.5	3.2	139.3	–	2.8	2.8	0.28	0.28
Average			1010.8		68.4	73.0	11.5	11.4	3.6	3.2	188.4		1.6	1.3	0.49	0.44

Data for atmospheric pressure and global radiation were missing for the city of Malmö

*P* Atmospheric pressure, *RH* relative humidity, *T* air temperature, *u* wind speed, *R* global radiation, *Precip.* precipitation, *VPD* vapour pressure deficit

In Malmö, groups were less coherent. The dry LWTs (A, NE, E and SE with VPD > 0.44 kPa) differed mainly in wind speed; LWTs E and SE were considerably windier than A and NE (Table 1 and Fig. 1c). LWT SW was associated with warm, calm and low VPD conditions, but also with rainfall (2.9 mm) exceeding the daily average (1.3 mm day^−1^) together with LWTs W and C. In Malmö, precipitation was in general lower than in Gothenburg and LWTs were on average cooler, calmer and contrasted less in temperature between types (Table 1). Thus, LWTs varied less in meteorological properties in Malmö than in Gothenburg. This dissimilarity is linked to the geographic differences in location and surrounding region affecting the air mass properties. The fact that less rain fell in Malmö is likely due to the presence of the large European continental landmass in the south-westerly direction, over which wet and windy Atlantic air masses commonly are transported. The humidity of these air masses may precipitate to a large degree before their arrival in Malmö. Similarly, the lower average wind speed in Malmö can be explained by the larger friction imposed on the air masses when they move from west and south-west over the continental landmass, as compared to when they move over the sea towards Gothenburg.

In most studies, relative humidity is the most commonly used measure of the dryness of the air, which governs anther burst, pollen release and atmospheric pollen dispersal (Bunderson and Levetin 2014). In this study, we have shown (see Sect. 3.6) that the quantitative measure for air dryness, VPD, had stronger relationships with response variables in comparison with RH. Few studies focus on the VPD influence on pollen (Aylor 2003; van Hout et al. 2008) and the inclusion of VPD could for example benefit the modelling of pollen emission and atmospheric dispersal (Schueler and Schlüntzen 2006) as a mechanistically more sound measure of the drying power of air.

### Birch pollen levels in different LWTs

**Fig. 2:**
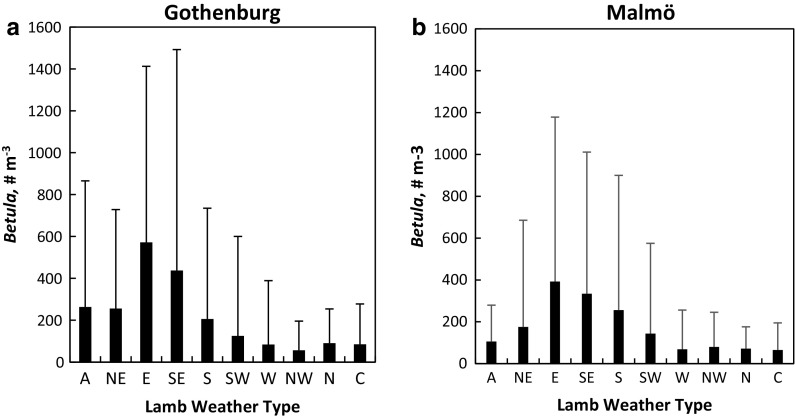
*Bars* show the averages and standard deviations of daily atmospheric birch (*Betula*) pollen concentrations in Gothenburg (**a**) and Malmö (**b**) for different LWTs during the birch pollen seasons 2006–2012

Since LWTs differed in meteorological characteristics (Table 1), it could be expected that certain LWTs are more strongly associated with high levels of airborne birch pollen. The dry and warm LWTs E and SE were associated with the highest daily averages of birch pollen in both cities (Fig. 2 and Table 1). High standard deviations, indicating that high pollen concentrations sometimes occurred, were also found for LWTs A, NE, S and SW in Gothenburg and LWTs NE, E, SE, S and SW in Malmö. The lowest averages were found for the generally wet or windy LWTs W, NW, N and C, with the addition of LWT A in Malmö only.

On average, conditions characterized by dry, warm and low/moderate wind speeds tend to promote high ambient pollen levels (Laiidi 2001; Khwarahm et al. 2014). In line with this, on the whole, the first hypothesis was valid for birch pollen concentrations; high levels were mainly associated with LWTs characterized by dry and calm to moderate conditions e.g., LWTs E and SE. However, the average pollen concentrations were lower in Malmö than in Gothenburg. The region immediately north, east and south of Malmö consists to a large degree of farmland (Germundsson and Schlyter 1999) with only isolated stands of birch trees (Nilsson 1990). In contrast, young birch trees are very common in green areas within the city of Gothenburg and in forest areas surrounding the city. Nearby sources of birch trees are expected to contribute substantially to local pollen concentrations (Faegri and Iversen 1989) and the smaller density of birch populations in the Malmö region partly explains the lower pollen concentrations as compared to Gothenburg. Birch pollen is a local to regional scale aerosol, due to its relatively short atmospheric lifetime from tens of hours to a few days (Sofiev et al. 2013), but long-range transport can add to the local birch pollen concentrations (Ranta et al. 2006; Skjøth et al. 2007). Because of the weaker local/regional source strength in Malmö, long-range transport of pollen is probably of larger significance for the magnitude of the birch pollen concentrations, than in Gothenburg where both local/regional and long-range transported birch pollen contribute substantially to registered pollen levels.

### Air pollution variation in relation to LWTs during the birch pollen season

**Fig. 3:**
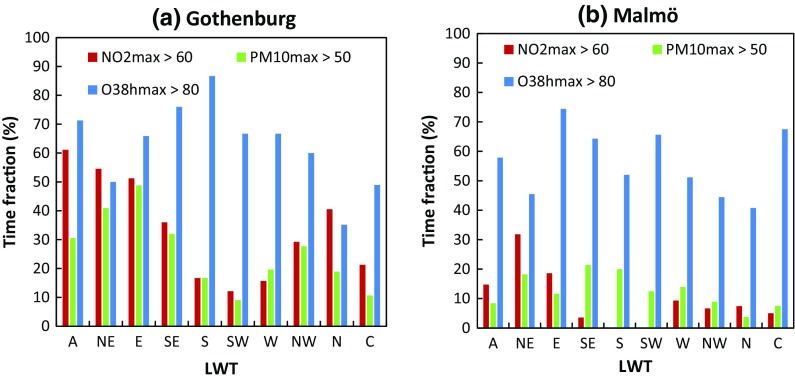
*Bars* showing the time fractions for each LWT of air pollutants exceeding daily maximum thresholds (NO_2 max_ > 60 µg m^−3^, PM_10 max_ > 50 µg m^−3^ and O_3 8h max_ > 80 µg m^−3^) in Gothenburg (**a**) and Malmö (**b**) during the birch pollen season

The exceedances of daily air pollutant thresholds were not equally common in different LWTs. In Gothenburg, weather types were more clearly differentiated in this regard than in Malmö (Fig. 3). In Gothenburg (Fig. 3a), high [NO_2_] and [PM_10_] occurred especially often in LWTs A, NE, E and SE (36–61% of the time), with the addition of N for [NO_2_]. In contrast, high levels were relatively uncommon (9–21%) in LWTs S, SW, W and C. Occurrences of high ozone levels varied distinctly over weather types, high levels were most common in LWT A, SE and S (71–87% of the time), and least common in N (35%). In Malmö, high [NO_2_] and [PM_10_] did not occur frequently in most LWTs (Fig. 3b), but high [NO_2_] were most common in LWTs A, NE and E (18–33%). High [PM_10_] were most frequent in LWTs NE, SE and S (18–21%) and lowest in A, NW, N and C (4–9%). High [O_3_] in Malmö were most common in LWT E, SE, SW and C (64–74%), and least common in NE, NW and N (41–45%; Fig. 3b).

Calm to moderate wind speeds are known to promote high levels of NO_2_ and PM_10_ (Grundström et al. 2015b; Jones et al. 2010). The fact that many of such LWTs in this study were also dry (low precipitation and high VPD) further promoted high PM_10_. The first hypothesis was supported for [NO_2_] and [PM_10_] in Gothenburg, which reached high daily maximum levels most often in dry and moderately calm LWTs.

### Covariation between high exposure situations for pollen and air pollution

**Fig. 4:**
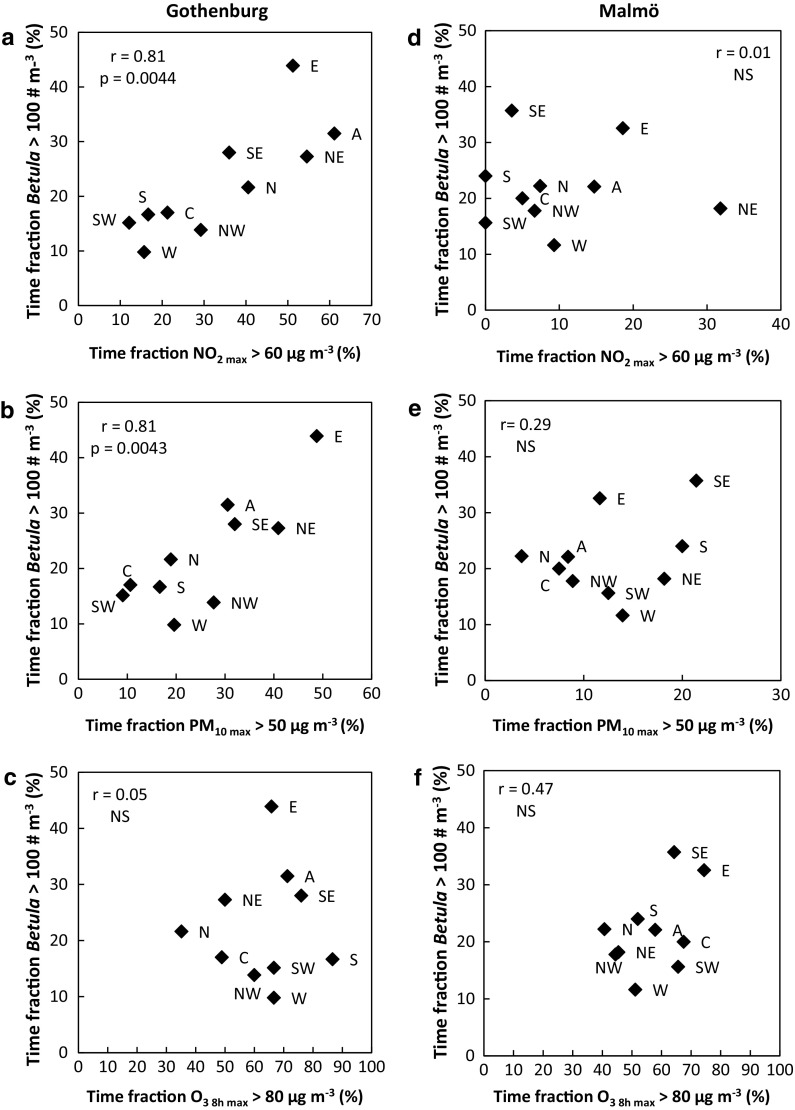
Relationships between time fractions of high daily atmospheric birch (*Betula*) pollen concentrations and exceedances of daily maxima of [NO_2_]_max_, [PM_10_]_max_ and [O_3_]_8h max_ in Gothenburg (**a**–**c**) and Malmö (**d**–**f**). The strength of the relationships was determined using Pearson product correlation coefficient (*r*) and significance level, using the *F*-test with *p* values referring to: *p < 0.05, **p < 0.01, ***p < 0.001, *NS* not significant

Within each LWT, high levels of a certain aerosol occurred in a specific fraction of time (Fig. 4). We analysed whether the specific time fractions of high daily birch pollen concentration (>100 pollen m^−3^) were correlated with those of high air pollution levels. A strong and significant relationship (*r* = 0.81; *p* = 0.0044) was found between the occurrence of high birch pollen and high daily NO_2_ maxima ([NO_2_]_max_ > 60 µg m^−3^) in Gothenburg (Fig. 4a). There was also a strong and significant (*r* = 0.81; *p* = 0.0043) positive correlation between the occurrence of high birch pollen levels and [PM_10_]_max_ > 50 µg m^−3^ (Fig. 4b). High levels of both aerosols were most common during LWTs A, NE, E and SE, indicating a similar response of pollen and air pollution to the meteorological conditions of these LWTs. In Malmö (Fig. 4d–f), no significant relationship was found between high daily birch pollen concentrations and daily maxima of [NO_2_] nor of [PM_10_]. This is partly explained by the smaller differences between LWTs in meteorological characteristics in Malmö, reducing distinct LWT effects on processes governing high air pollution. Also the lower traffic amount in Malmö, being a smaller city than Gothenburg, causes smaller air pollution emissions and hence lower pollution levels in general. Additionally, the close vicinity to Copenhagen likely affects air pollution levels in the Malmö region. Furthermore, as mentioned in Sect. 3.2, birch pollen levels are potentially more influenced by long-range transport in Malmö, originating from sources far away from the air pollution sources. All these factors add noise and complicate the weather effects on pollen/pollution levels, thus explaining the relatively poor covariation between high birch pollen with both [NO_2_]_max_ and [PM_10_]_max_ in Malmö.

There were no significant relationships between the occurrence of high daily birch pollen concentrations and of high daily ozone levels (O_3 8h max_ > 80 µg m^−3^) in any of the cities. In both cities, at least 40% of the daily maxima of [O_3_] reached high levels during most LWTs, apart from LWT N in Gothenburg.

### OTC antihistamines sold in relation to pollen and air pollution

**Fig. 5:**
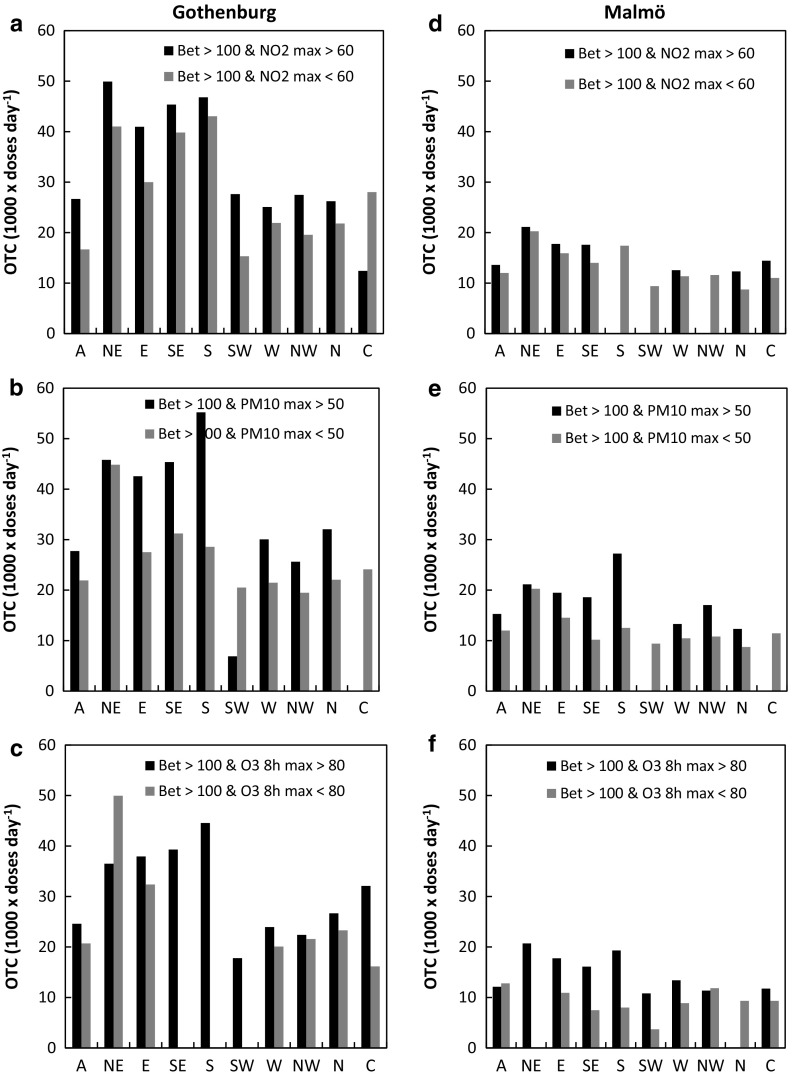
Sales of OTC antihistamines during high daily atmospheric birch pollen concentrations and high/low daily air pollution (NO_2_, PM_10_, O_3_). All data are during birch pollen seasons in Gothenburg (**a**–**c**) and Malmö (**d**–**f**), 2006–2012

From a public warning perspective, it is important to know which situations are associated with high exposure to pollen and air pollution, both simultaneously and individually. As a proxy for allergy symptoms, we used the demand for antihistamines, expressed as sold over-the-counter (OTC) defined daily doses (DDD), formerly used by, e.g., Fuhrman et al. (2007), Caillaud et al. (2015) and Sheffield et al. (2011). The effect of simultaneously high birch pollen and air pollution concentrations was investigated by comparing the daily numbers of OTC antihistamine doses sold during high birch pollen/low air pollution to high birch pollen/high air pollution. For a majority of LWTs in both Gothenburg (23 LWTs out of the 30 in total) and Malmö (21 LWTs out of the 30 in total), the OTC sales of antihistamines were higher during situations when high birch pollen levels coincided with high concentrations of air pollutants (black bars in Fig. 5a–f, respectively). The highest relative OTC sales in both cities occurred in LWTs NE, E, SE and S. In four cases in Gothenburg and five cases in Malmö, out of the 30, this comparison could not be made since high pollen levels were always only associated with either high or low air pollution.

**Fig. 6:**
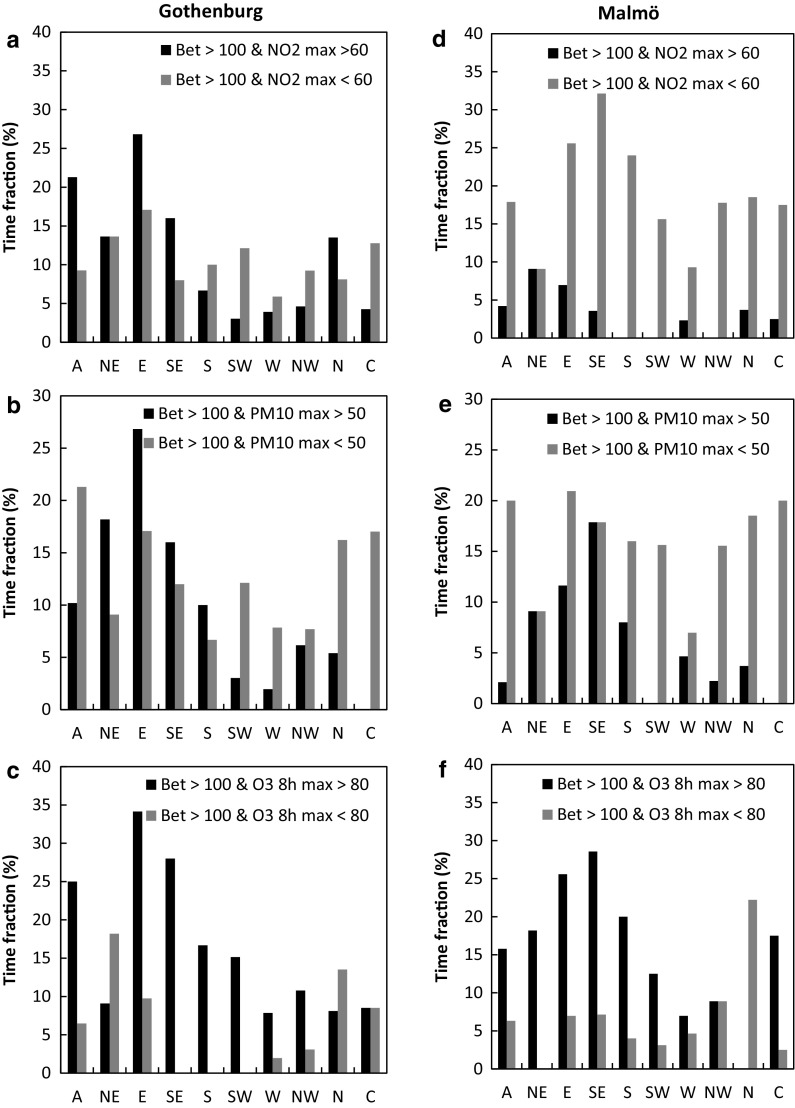
Time fractions of simultaneously high daily atmospheric birch pollen concentrations and high/low daily air pollution (NO_2_, PM_10_, O_3_). All data are during birch pollen seasons in Gothenburg (**a**–**c**) and Malmö (**d**–**f**) 2006–2012

The occurrence of days with simultaneously high air pollution ([NO_2_]_max_ and [PM_10_]_max_) and high birch pollen was overall more frequent in Gothenburg (Fig. 6a–c) than in Malmö (Fig. 6d–f). In Malmö, these situations only occurred one day in some LWTs (for [NO_2_]_max_ LWTs SE, W, N and C and for [PM_10_]_max_ LWT NW and N). Since the overall air pollution load was smaller in this city, and more so during the LWTs mentioned above, it can be expected that days with simultaneous high levels occur more rarely. For certain weather types in Malmö simultaneous high levels did not occur at all (for [NO_2_]_max_ LWTs S, SW and NW, for [PM_10_]_max_ LWTs SW and C and N for O_3 8h max_). Co-exposure to high levels of birch pollen and of air pollution ([NO_2_]_max_ and [PM_10_]_max_; 3 and 6% of the time on average, respectively, across LWTs) were lower in occurrence in comparison with high levels of birch pollen and low levels of air pollution (19 and 17% of the time on average, respectively, across LWTs). Thus, despite the fewer situations with simultaneously high levels, these had larger OTC sales for a majority of the LWTs (7 out of 10 LWTs for high birch pollen levels and [NO_2_]_max_ and 8 out of 10 LWTs for high birch pollen levels and high [PM_10_]_max_). This result further highlights the likely enhancing effect on pollen symptoms by air pollution (NO_2_ and PM_10_) in Malmö. Simultaneous high birch pollen and high ozone levels ([O_3_]_8h max_ > 80 µg m^−3^) were more common than high birch pollen and low ozone levels ([O_3_]_8h max_ < 80 µg m^−3^) most of the time (7 out of 10 LWTs in Gothenburg and 8 out of 10 LWTs in Malmö). Higher OTC sales were observed for simultaneous high population exposure for birch pollen and for air pollution (daily [NO_2_]_max_ and [PM_10_]_max_), despite their lower occurrence, that was observed for days with high birch pollen and low air pollution levels. Thus, the second hypothesis was supported for birch pollen concentrations, [NO_2_]_max_ and [PM_10_]_max_. In Malmö, this effect was more obvious than in Gothenburg.

### Correlation analysis of birch pollen, air pollution and OTC with meteorological variables on an LWT basis

For each LWT, average meteorological variables were correlated with average birch pollen concentrations, OTC, time fractions of high birch pollen concentrations (f Bet > 100) and simultaneously occurring high levels of birch pollen and air pollutants (f Bet > 100 and NO_2 max_ > 60, f Bet > 100 and PM_10 max_ > 50, f Bet > 100 and O_3 max_ > 80), as shown in Table 2. In Gothenburg, it was obvious that the VPD and temperature in LWTs explained a substantial part of the variation in the average values of birch pollen and OTC, with strong and significant positive relationships (*r* ≥ 0.8). Furthermore, time fractions of high birch pollen and simultaneously high levels of birch pollen and air pollutants had very strong and significant relationships with VPD. RH also showed some strong relationships, but in general, VPD, the more mechanistically based measure of air dryness, was superior to RH. Average wind speeds in LWTs only related weakly to the tested response variables.

**Table 2:** Statistics for the correlation analysis between meteorological variables [averages of vapour pressure deficit (VPD), relative humidity (RH), temperature (*T*) and wind speed (*u*)] and response variables (averages of *Betula* and OTC and time fractions (f) of; Bet > 100, Bet > 100 and NO_2 max_ > 60, Bet > 100 and PM_10 max_ > 50, Bet > 100 and O_3 max_ > 80

Response variables	VPD		RH		*T*		*u*	
	*r*	Sign	*r*	Sign	*r*	Sign	*r*	Sign
Gothenburg								
*Betula*	**0.90**	***	**0.82**	**	**0.94**	***	0.45	NS
f Bet > 100	**0.92**	***	**0.97**	***	**0.90**	***	0.61	NS
f Bet > 100 and NO_2 max_ > 60	**0.93**	***	**0.92**	***	**0.82**	**	0.59	NS
f Bet > 100 and PM_10 max_ > 50	**0.94**	***	**0.92**	***	**0.88**	***	0.42	NS
f Bet > 100 and O_3 max_ > 80	**0.80**	**	0.71	*	**0.82**	**	0.44	NS
OTC	**0.82**	**	**0.83**	**	0.75	*	0.43	NS
Malmö								
*Betula*	0.62	NS	0.51	NS	0.65	*	0.05	NS
f Bet > 100	0.54	NS	0.47	NS	0.57	NS	0.16	NS
f Bet > 100 and NO_2 max_ > 60	0.67	*	0.58	NS	0.27	NS	0.60	NS
f Bet > 100 and PM_10 max_ > 50	0.59	NS	0.50	NS	0.48	NS	0.15	NS
f Bet > 100 and O_3 max_ > 80	0.53	NS	0.36	NS	0.53	NS	0.12	NS
OTC	**0.82**	**	0.77	**	0.46	NS	0.23	NS

All relationships are based on averages or time fractions for each LWT and thus consist of 10 points. The strength of the relationship was determined using Pearson product correlation coefficient (*r*) and significance level, using the *F*-test with *p* values referring to: * *p* < 0.05, ** *p* < 0.01, ***p  < 0.001, *NS* not significant

The bold correlation coefficients indicate a strong relationship with a value equal to or above 0.80

In Malmö, the only strong and significant relationship was found between VPD and OTC (*r* = 0.82, Table 2). All other meteorological variables related more or less weakly to the tested response variables. The smaller contrast between meteorological characteristics of Malmö LWTs leads to a weak distinction of atmospheric processes which govern either accumulation or dilution of aerosols. In Gothenburg, the meteorological characteristics of LWTs were clearer (Fig. 1 and Table 1), thus more efficiently separating accumulation-governing processes from dilution processes and producing stronger relationships between response variables and meteorological variables.

## Concluding summary

This study has shown that airborne birch pollen concentrations varied closely in relation to LWTs in both cities. Highest levels were found in LWTs E and SE. There was a distinct pattern with frequent high air pollution levels in relation to certain LWTs in Gothenburg, but the relationship was not as clear for Malmö, where pollution levels were lower. OTC sales were high during high pollen LWTs (NE, E, SE and S) in both cities. Ozone levels were generally high during the birch pollen season and often exceeded the threshold value during most LWTs, but, unlike birch pollen, NO_2_ and PM_10_ did not exhibit a strong pattern with respect to LWTs. Furthermore, the meteorological characteristics of the LWTs could be used to explain a large fraction of the variation in many response variables in Gothenburg. In Malmö, this was seldom the case, which is likely due to the less contrasting properties of the LWTs, together with a smaller load of air pollution as well as lower levels of locally derived birch pollen.

The citizen’s right to information on environmental factors that may have an adverse impact on health and life quality makes it possible to take preventive measures, which is identified in a number of EU directives (Karatzas 2009; Karatzas et al. 2013). Furthermore, it was recently pointed out that, as evidence accumulates on the joint effects of biological, chemical and physical (meteorological) factors, the need for an integrated approach to the assessment, forecasting, and communication of air quality is ever more apparent (Klein et al. 2012). To this end, the results of the present study are a contribution.

The most important conclusions from this study were:

- The two cities differed in birch pollen level (Gothenburg > Malmö), in air pollution level (Gothenburg > Malmö for NO_2_ and PM_10_ but not O_3_) and in meteorological characteristics associated with LWTs. LWT characteristic differences were smaller in Malmö than in Gothenburg, e.g., with respect to VPD and precipitation.
- There was a strong contrast between different LWTs in birch pollen levels, easterly and southerly LWTs having higher levels in both cities.
- Air pollution levels differed strongly between LWTs, especially for NO_2_ and PM_10_ in Gothenburg.
- In Gothenburg, there was a strong covariation between high birch pollen and high NO_2_/PM_10_.
- Sales of OTC antihistamines were higher in 70–90% of the LWTs during situations with simultaneous high birch pollen and high air pollution levels, compared to situations with high birch pollen coinciding with low air pollution levels.
- Even in LWTs for which simultaneously high birch pollen and high air pollution levels were rare, the results related to OTC antihistamines sold in Malmö indicate that air pollution worsened the effects of symptoms.
- The variation of VPD and T among LWTs in Gothenburg showed strong positive relationships with all tested response variables. High VPD and T indicate two important characteristics of the combined meteorology to promote high levels of pollen and air pollutants which were typical for LWTs NE, E, SE and S. As a measure of air dryness, VPD was superior to RH.
- The information obtained in this study with respect to covariation of pollen, air pollutants and weather can be useful in risk management and information systems.
